# Personality traits differentially affect components of reproductive success in a Neotropical poison frog

**DOI:** 10.1098/rspb.2023.1551

**Published:** 2023-09-20

**Authors:** Mélissa Peignier, Yimen G. Araya-Ajoy, Max Ringler, Eva Ringler

**Affiliations:** ^1^ Division of Behavioural Ecology, Institute of Ecology and Evolution, University of Bern, 3032 Hinterkappelen, Switzerland; ^2^ Messerli Research Institute, University of Veterinary Medicine Vienna, 1210 Vienna, Austria; ^3^ Centre for Biodiversity Dynamics, Department of Biology, Norwegian University of Science and Technology, 7034 Trondheim, Norway; ^4^ Department of Behavioral and Cognitive Biology, University of Vienna, 1030 Vienna, Austria; ^5^ Department of Evolutionary Biology, University of Vienna, 1030 Vienna, Austria; ^6^ Institute of Electronic Music and Acoustics, University of Music and Performing Arts Graz, 8010 Graz, Austria

**Keywords:** mating success, offspring production, selection gradients, aggressiveness, exploration, boldness

## Abstract

Individual reproductive success has several components, including the acquisition of mating partners, offspring production, and offspring survival until adulthood. While the effects of certain personality traits—such as boldness or aggressiveness—on single components of reproductive success are well studied, we know little about the composite and multifaceted effects behavioural traits can have on all the aspects of reproductive success. Behavioural traits positively linked to one component of reproductive success might not be beneficial for other components, and these effects may differ between sexes. We investigated the influence of boldness, aggressiveness, and exploration on the number of mating partners, mating events, and offspring surviving until adulthood in males and females of the Neotropical poison frog *Allobates femoralis*. Behavioural traits had different—even opposite—effects on distinct components of reproductive success in both males and females. For example, males who displayed high levels of aggressiveness and exploration (or low levels of aggressiveness and exploration) managed to attract high number of mating partners, while males with low levels of boldness, low levels of aggressiveness, and high levels of exploration had the most offspring surviving until adulthood. Our results therefore suggest correlational selection favouring particular combinations of behavioural traits.

## 1. Introduction

Individual reproductive success is determined by many factors, such as the number of mating events, the number of mating partners, produced offspring, and offspring survival until adulthood [1]. Animal personality—behavioural differences between individuals that are consistent across time and context—has been shown to impact on an individual's likelihood to obtain mating partners and its chances to survive until the next breeding season, and thereby influence individual reproductive success [2–5]. Previous studies revealed that personality influences single components such as the number of offspring that were produced or survived to adulthood, or parental performance [6–8]. For example, fast-exploring female blue tits (*Cyanistes caeruleus*) have higher reproductive success than slow-exploring females because they feed their offspring more frequently [9]. Though previous research provided empirical evidence for the impact single personality traits have on specific components of reproductive success, we need to learn more about the effects that combinations and interactions of personality traits have on the various processes that shape reproductive success in males and females.

Several hypotheses have been proposed to explain why individual behavioural differences emerge and are maintained [10]. The one that previously received the strongest support states that behavioural differences between, and behavioural consistency within, individuals are linked to life-history trade-offs [11,12]. For example, more aggressive or bold males might more successfully reproduce within a single season but suffer from decreased survival, while less aggressive or shy males might have fewer mates and offspring annually, but live to reproduce over more seasons.

Previous studies mainly linked single personality traits with one measure of reproductive success or survival (e.g. [7,13–16]). However, personality traits that enhance one component of reproductive success might not be as beneficial to another. For example, individuals that are bolder and more aggressive might benefit from being more visible to mates and better able to defend a territory, but be less dedicated parents, which might reduce their offspring's chances of survival. Unfortunately, only few studies have sought to determine the contrasting effects personality traits may have on the different components of reproductive success (e.g. acquisition of multiple mating partners, number of offspring produced, and offspring survival; but see [3]). Furthermore, correlational selection may favour different combinations of personality traits [17], which typically occurs when the influence one personality trait exerts on a component of reproductive success depends on another personality trait score [18]. Selection then maintains phenotypic correlations among traits; for instance, aggression might only increase reproductive success if individuals are also bold. In addition, the effect of a particular personality trait on reproductive success may differ between males and females, because selective pressure on each sex's reproductive behaviours may differ [19,20].

Amphibians are an ideal taxon to study links between behaviour and reproductive success, as they feature diverse reproductive behaviours [21–23]. In particular, Neotropical poison frogs (Dendrobatidae *sensu* [24]) have ideal prerequisites for within- and between-individual behavioural variation, including territoriality, elaborate courtship behaviour, and complex parental care [25–32]. In this study, we thus monitored a free-ranging population of the brilliant-thighed poison frog (*Allobates femoralis*) during their reproductive season to identify the influence of personality traits on the various processes that shape reproductive success.

In male and female *A. femoralis*, we specifically assessed the influence of boldness, aggression, and exploration levels on number of clutches sired/produced (i.e. number of mating events), number of mating partners, and ‘number of adult offspring’. We conducted repeated behavioural experiments and collected tissue samples for genetic analysis to infer parent–offspring relationships. We hypothesized that aggressiveness, boldness, and exploration have non-independent and sex-specific effects on the different components of reproductive success. We thus explored the possibility of correlational selection measured as interactive effects of different behavioural traits on fitness. In summary, we found support for our prediction that personality traits differentially influenced the three components of reproductive success, which we argue could lead to the evolution of different reproductive strategies.

## 2. Methods

### (a) Study system

*Allobates femoralis* is a diurnal frog with a highly promiscuous mating system [33–35]. Studies on animal personality in this species found that males and females display personality and vary in their levels of exploration and boldness; males also vary in their aggression level [36,37].

During the reproductive season, males are highly territorial and announce territory occupancy via a prominent advertisement call to repel male competitors and attract females [38,39]. Typically, most males in the population manage to establish a fixed territory, and only few individuals switch the location of their territory over the season. The possession of a territory is a prerequisite for mating success in males [35]. To mate, females commute from their perching site to male territories within a 20 m radius and typically decide with whom to mate before they approach a male [34,40,41]. Females lay clutches in the leaf litter inside the male's territory and after 15–21 days of larval development males transport the newly hatched tadpoles to water bodies outside their territory [42–45].

### (b) Study site and population survey

Our study was conducted on an experimental population of *A. femoralis* on a river island of about 5 ha in a Neotropical lowland rainforest (T1.1 *sensu* [46]). The island is in the Les Nouragues nature reserve in French Guiana, near the Saut Pararé field camp of the CNRS Nouragues Ecological Research Station (4°02′ N, 52°41′ W [47]). The population was introduced in 2012 and has since comprised about 150 adults (for detailed information see [45]). As the island is surrounded by a fast-flowing river and *A. femoralis* is strictly terrestrial, our experimental population can be considered naturally confined, with no possible em- or immigration of adults between the island and a nearby mainland population.

Between February and April 2019, we monitored the population on the island from 09.00 to 18.00 each day and caught every adult frog we encountered. We visually identified all frogs via their distinct ventral patterns, and further confirmed their identity with the pattern-matching software Wild-ID [48]. We sexed frogs by the presence (males) or absence (females) of vocal sacs. We collected tissue samples for genetic analysis from all newly encountered adults by removing the third toe from both hind limbs, immediately preserving it in 96% ethanol [49]. At each capture, we digitally mapped the frog's spatial location in the mobile GIS software ArcPad 10 (ESRI, Redlands, CA, USA) installed on rugged Win10 tablets (CAT T20, Bullitt Group, Reading, UK) [50] and then handled the data in ArcGIS 10.6 (ESRI). As females are usually harder to detect than males, we ensured we sampled most of the population by calculating the sampling coverage for both sexes from asymptotic population size estimates (MMMeans [51]) in EstimateS 9.1 [52].

Since 2018, frogs in the study area usually deposit tadpoles in an array of 14 artificial pools (volume: approx. 15 l). As the island features very few suitable natural deposition sites, these artificial pools are the main resource used for tadpole deposition in *A. femoralis*. To emulate natural pools, which are typically ephemeral and often change, we removed the 14 artificial pools after two weeks and opened 16 pools (volume: approx. 5 l) in new locations. Between February and May 2019, we regularly sampled tadpoles from the pools, before and after we changed their location. We collected tissue samples from tadpoles by clipping the tip of their tail, preserving it in 96% ethanol. We released tadpoles in artificial pools after clipping so they could continue to mature. We returned in 2020, from February to mid-March, to collect tissue samples from the next cohort of adult frogs and used these to calculate the number of offspring from 2019 that survived to adulthood (see below, Parentage analysis).

### (c) Behavioural tests

For the present study, we used data and results from a previous study investigating levels of aggressiveness, boldness, and exploration in the same *A. femoralis* population [37]. This earlier study had been conducted concurrently to the parentage analysis presented in this study. It measured levels of territorial aggression using acoustic playbacks to evoke territorial defence behaviour in focal males (but not in females, who do not exhibit territorial aggression). It also assessed individual levels of exploration and boldness in both sexes, using a novel environment test. In total, 163 territorial defence tests were performed and included 51 males (mean ± s.d. = 3.20 ± 1.31 repetitions per individual) and 238 novel environment tests were performed and included 52 males and 35 females (mean ± s.d. = 2.74 ± 1.33 repetitions per individual) [37].

Results from this previous study showed that the behaviours measured in both tests were repeatable. In addition, using structural equation modelling, this previous study described the structure of the behaviours measured into three functional units, effectively finding evidence for the prevalence of the personality traits aggressiveness (only in males), exploration, and boldness. Based on results from the repeatability analysis and structural equation models, we chose in the current study the three behaviours that best represented the personality traits aggressiveness, boldness, and exploration. These behaviours were the latency to jump towards an intruder in the territorial defence test, the time spent in the shelter, and the distance travelled in the novel environment test, respectively (for more details see [37]). We refer to the measures of these three behaviours as personality ‘scores’ in the rest of the paper.

### (d) Parentage analysis

We used molecular parentage analysis to determine the number of clutches, number of mating partners, and number of adult offspring produced by the adult males of 2019. Since the island's population had been monitored over the long term since 2012, we also had access to DNA of older individuals (survivors of previous years, encountered again in 2019). In 2019, we sampled 121 adults (64 males and 57 females; hereafter ‘adults from 2019’), and 1142 tadpoles (hereafter ‘tadpoles from 2019’). In 2020, we sampled 71 adults (27 males and 44 females), of which 55 were new encounters (descendants of the 2019 cohort, hereafter ‘adult descendants from 2019’).

To isolate genomic DNA from the tissue samples, we performed a Proteinase K digestion immediately followed by an extraction with a DNeasy kit (QIAGEN, Valencia, CA). We then used fluorescent-labelled primers and PCR protocols to amplify microsatellites at twelve highly variable loci (Afem03, Afem04, Afem05, Afem09, Afem12, Afem13, Afem16, Afem20, Afem22, Afem24, Afem25, Afem27) [53,54]. The amplified products were diluted with water and mixed with internal size standard LIZ, then run on a capillary sequencer (ABI 3730, Applied Biosystems/Thermo Fisher Scientific, Waltham, MA, USA). We visually identified all loci and determined raw allele size estimates with the software PeakScanner 1.0 (Applied Biosystems). We used the binning software Tandem 1.01 [55] to determine final allele sizes. Individuals were excluded from further analyses when we failed to score four or more loci. Ultimately, we reconstructed the pedigrees of 57 male and 53 female adults from 2019, 1109 tadpoles from 2019, and 55 adult descendants from 2019.

We used COLONY 2.0.6.7 software for the parentage analysis [56], building a medium-precision full likelihood model that allowed for polygamous mating in both sexes, without setting a sibship prior. We determined parent–offspring relationships by treating individual adults from 2019 as potential ‘fathers’ and ‘mothers’, and treating all tadpoles from 2019 as potential ‘offspring’. To further investigate the influence of personality on number of adult offspring of the adult males from 2019, we treated all adults from 2019 as potential parents; we treated all adult descendants from 2019 as potential ‘offspring’. We used ‘Best (ML) Configuration’ for the analysis; the software simulated parental genotype when it could not identify one or both parents of a tadpole. Of the 1109 tadpoles from 2019, COLONY assigned 1006 (90.7%) to at least one known parent; 695/1006 (69.1%) had both parents identified. Of the 2019 group, 52/57 (91.2%) of adult males and 47/53 (88.7%) of adult females were assigned to at least one tadpole; 47 males (85.5%) and 48 females (87.3%) were assigned to at least one tadpole that reached adulthood in 2020. All 55 adult descendants from 2019 were assigned to at least one known parent.

From these parentage assignments, we determined the number of clutches and the number of mating partners for each adult male and female in 2019. Because males distribute their clutches across several pools [57] and captive *A. femoralis* females lay an average of 1 clutch every 8 days [58], we assumed that tadpoles from an identified pair that were deposited in one or more pools less than 6 days apart belonged to the same clutch; these represented one mating event. If a group of tadpoles had been deposited on a given day and was assigned to only one known parent, we considered them to have originated from one clutch of this known parent and a new, unknown mate. We assumed tadpoles from the same mating pair originated from two separate clutches if the interval between the depositions was more than 6 days. If only one parent was assigned to two clutches deposited more than 6 days apart, we assumed that two different mating pairs produced the clutches. Finally, to count the number of adult descendants from 2019, we only used parent–offspring triads for which at least one parent was identified.

### (e) Statistical analysis

All statistical analyses were performed in R v3.6.0 [59]. Each parent in our sample was characterized by the total number of clutches, mating partners, and adult offspring obtained. We assessed the influence of personality traits on reproductive success separately for males and females to avoid artificially increasing sample size due to clutches being assigned to a father and a mother, and because we had different expectations for both sexes. We also ruled out a potential confounding effect by investigating the prevalence of (dis)assortative mating based on personality (we present methods and results in the electronic supplementary material). An earlier study found that in *A. femoralis,* body size was unrelated to mating or reproductive success [35], so we did not include body size in our statistical analyses.

We investigated the influence of personality traits on male and female reproductive success. First, we extracted the best linear unbiased predictors (BLUPs) of the expected personality values from random intercept models with either aggressiveness, boldness, or exploration as response variable and ID as random effect, for each sex separately. We then used the ‘lme4’ package [60] to build three generalized linear models (GLMs) for each sex; with number of clutches, number of mating partners, or number of adult offspring as response variables. In the models focusing on females only, we added an interaction between the BLUPs (scaled by subtracting the mean and dividing by the standard deviation) of boldness and exploration as fixed effects. In the male models, we used as fixed effect an interaction between the scaled BLUPs of aggression, boldness, and exploration scores. Because having more mating partners automatically relates to having more clutches—one more mate identified from offspring corresponds to at least one more clutch sired—we included the number of different mates as a fixed effect in the models where the response variable was the number of clutches. In the models where the response variable was the number of adult offspring, we included as fixed effects the number of different mates and number of clutches. Adding these variables as fixed effects in the models is essentially similar to fitting a path analysis [61,62] and enabled us to study the direct and indirect effects of behaviours on each of the components of reproductive success. All models assumed a Poisson error distribution and were checked for overdispersion. Models with the number of adult offspring as response variable were slightly over-dispersed in both males and females, we thus fitted negative binomial models. Since models followed a Poisson or a negative binomial distribution, our estimates of the relationship between phenotype and reproductive success closely approximated selection gradients [63].

BLUPs have the advantage of approximating the average behaviour, while taking into account the potential effect of the within-individual variance on the estimates of the means and the variance among means. However, several recent papers cautioned against misusing BLUPs in behavioural research [64,65]. In our study, we thus compared our results with error-in-variable models, which allow acquiring unbiased estimates of selection gradients and their uncertainty, while controlling for both measurement error and phenotypic plasticity [66,67]. We used Bayesian inference to estimate the joint likelihood of the path model parameters with the ‘rstan’ package [68]. We built two models (one per sex) that assumed a Poisson distribution for number of mating partners, number of clutches, and number of adult offspring. We added an interaction between boldness and exploration scores as fixed effects in the female model, and an interaction between aggressiveness, boldness, and exploration scores as fixed effects in the male model. In the models in which number of clutches was the response variable, we also included number of different mates as a fixed effect. In the models in which number of adult offspring was the response variable, we also included as fixed effects number of different mates and number of clutches. For both models, we ran 101 000 iterations with a burn-in of 1000, selecting every 100th posterior parameter sample after the initial burn-in. Because the models were highly complex, we used strong priors for the relationships that we knew (from the GLMs) should be positive (e.g. effect of some reproductive parameters on others). We also used informative priors for the means of the different components of reproductive success, but we used diffuse priors for the effects of behaviours on measures of reproductive success.

Error-in-variable models are data-hungry and credible intervals were large for some parameters in our dataset. Since the GLMs and the error-in-variable models returned estimates of comparable value (electronic supplementary material, tables S1, S2), we only present results from the GLMs in the main text. We provide point estimates and confidence intervals of the posterior distributions for the error-in-variable models in the electronic supplementary material (tables S1, S2).

## 3. Results

We sampled 57 female and 64 male *A. femoralis* in 2019. Population size estimates (MMMeans based on 1046 captures) predicted that the population comprised 76 females and 67 males, corresponding to a sampling coverage of 75% for females and 96% for males. On average, males had 2 different mating partners (range = 0–5), produced 3 clutches (range = 0–7), and had 1 offspring that survived until adulthood (range = 0–9). Females had on average 1 mate (range = 0–5), produced 2 clutches (range = 0–8), and had 1 offspring who survived until adulthood (range = 0–9). These results are similar to the findings of a previous study on a neighbouring population [35]. The number of adult offspring was higher in individual females with more mates and in individual males who sired more clutches (table 1).

**Table 1.** Results of the generalized linear models investigating the link between personality traits and different processes shaping reproductive success for males and females: model estimates, standard error, and *p*-values are presented. Based on [69], evidence of effects is reported with asterisks (i.e. 0.1 < *p*-value < 0.05: weak evidence*; 0.05 < *p*-value < 0.01: moderate evidence**; 0.01 < *p*-value: strong evidence***). Error-in-variable models are reported in electronic supplementary material, tables S1, S2.

	number of different mates			number of clutches			number of adult offspring		
	estimate	SE	*p*-value	estimate	SE	*p*-value	estimate	SE	*p*-value
*results for males (N = 51)*									
(intercept)	0.59	0.13	<0.001***	0.17	0.21	0.399	−3.62	1.20	0.003***
number of mates				0.38	0.07	<0.001***	0.02	0.34	0.952
number of clutches							0.57	0.24	0.018**
aggressiveness	−0.14	0.16	0.366	−0.20	0.14	0.138	0.27	0.39	0.485
boldness	−0.10	0.16	0.541	−0.04	0.15	0.807	−1.87	0.83	0.024**
exploration	−0.02	0.14	0.907	−0.07	0.11	0.530	0.52	0.51	0.303
aggressiveness × boldness	−0.19	0.23	0.396	−0.01	0.21	0.956	0.29	0.64	0.648
aggressiveness × exploration	−0.28	0.14	0.041**	−0.07	0.12	0.547	1.90	0.69	0.006***
boldness × exploration	0.04	0.14	0.792	−0.02	0.12	0.858	0.67	0.77	0.380
aggressiveness × boldness × exploration	−0.19	0.19	0.301	0.06	0.16	0.697	2.86	0.93	0.002***
*results for females (N = 36)*									
(intercept)	−0.17	0.26	0.505	−0.20	0.22	0.369	−1.05	0.65	0.103
number of mates				0.53	0.10	<0.001***	1.45	0.80	0.072*
number of clutches							−0.69	0.51	0.175
boldness	−0.40	0.24	0.095*	−0.24	0.17	0.162	−0.37	0.52	0.474
exploration	−0.88	0.36	0.015**	−0.07	0.26	0.799	−0.18	0.72	0.801
boldness × exploration	−0.53	0.20	0.010***	−0.00	0.16	0.988	0.44	0.60	0.462

**Figure 1.**
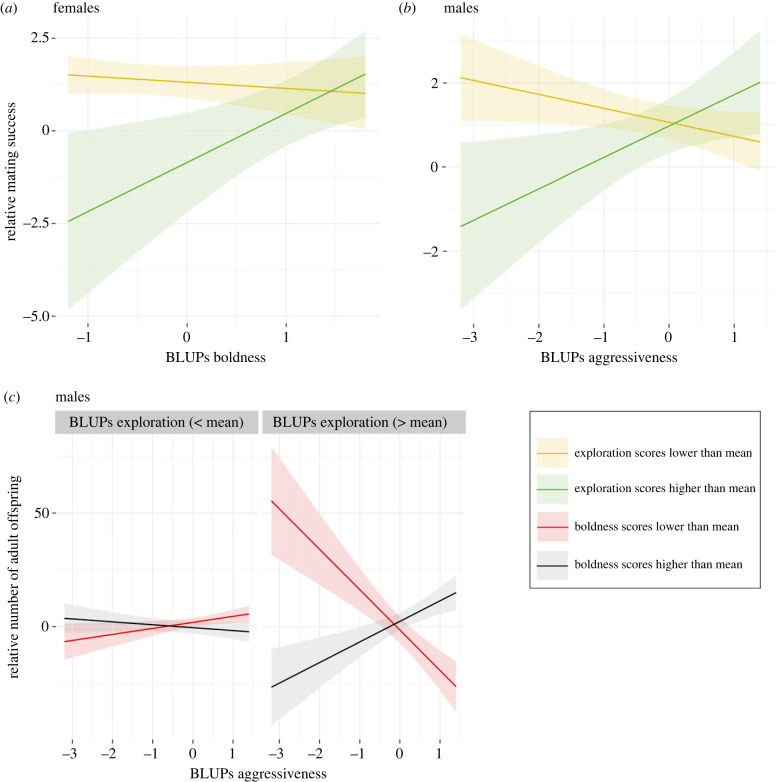
Influence of personality traits on components of reproductive success. This figure shows the marginal effect of interaction terms from the GLMs investigating (*a*) the link between exploration, boldness, and mating success in females, (*b*) the link between aggressiveness, exploration, and mating success in males, and (*c*) the link between aggressiveness, exploration, boldness, and number of adult offspring in males. To facilitate the visualization of the interaction effect, we split individuals in groups based on their personality scores. Yellow lines represent exploration scores lower than the population mean; green lines represent exploration scores higher than the population mean; red lines represent boldness scores lower than the population mean; and black lines represent boldness scores higher than the population mean. Areas around the lines present the 95% confidence intervals. The values for the phenotypes are BLUPs extracted from random regression models. BLUPs of aggressiveness and boldness were multiplied by −1 so that higher values represent higher levels of aggression and boldness. Reproductive success measures are relative, calculated by dividing each value by the mean population value, and show only the between-individual covariance between phenotype and reproductive success [65].

In males, we found moderate evidence (0.05 < *p*-value < 0.01, *sensu* [69]) that the interaction between aggression and exploration levels influenced the number of mates (table 1). Less aggressive males obtained more mating partners if their exploration level was low, while highly aggressive males obtained more mating partners if they were also highly explorative (figure 1*b*; electronic supplementary material, S1*b*). We also found strong evidence (0.001 < *p*-value < 0.01, *sensu* [69]) that exploratory behaviour influenced the number of mating partners in females (table 1). Females with low exploration scores, or with high exploration and high boldness levels obtained more mating partners (figure 1*a*; electronic supplementary material, S1*a*).

In females, personality did not influence the number of clutches produced (table 1) or the number of adult offspring. In males, we did find strong evidence that aggression, exploration, and boldness levels interacted to influence the number of adult offspring (table 1). Shy, non-aggressive males with high exploration levels had the most adult offspring (figure 1*c*). Bold, aggressive males with high exploration levels also had a high number of adult offspring (figure 1*c*). In males with low exploration levels, aggression and boldness levels had less effect on the number of adult offspring (figure 1*c*).

## 4. Discussion

**Figure 2.**
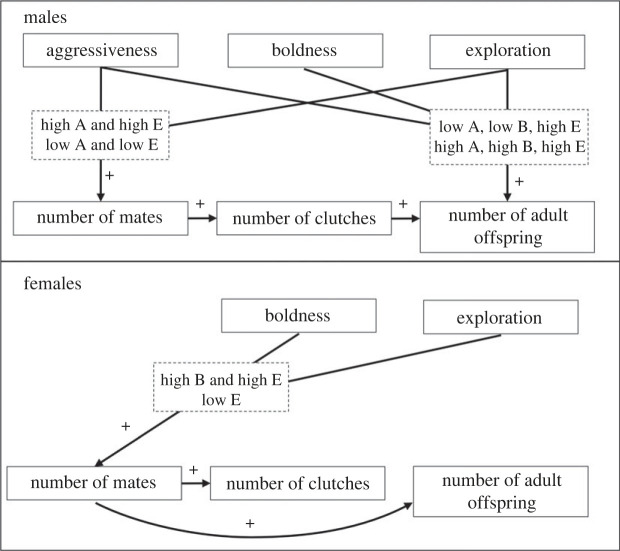
The influence of personality traits on components of reproductive success in males and females. Dashed squares contain the personality types with the highest value of the focal component of reproductive success; A is the level of aggression, B is the level of boldness, and E is the level of exploration.

We used a wild, free ranging population of *A. femoralis* to study how personality traits affect the various processes shaping reproductive success. We found that specific combinations of personality traits differently influenced components of reproductive success in both males and females (figure 2). Our results suggest that the effect of a personality trait on a component of reproductive success may be mediated by the level of other personality traits (figure 2).

Our path analysis allowed us to investigate the respective contributions of components of reproductive success in males and females. For males, the number of sired clutches was the main predictor for number of adult offspring in the following year, while females could increase the number of surviving adult offspring mainly by mating with multiple males. This finding is in contrast to the classical view of mainly males benefitting from multiple mating partners [70]. Despite empirical evidence of female polyandry in various animal taxa [71–74], the notion that mating with multiple partners is mainly beneficial to males still prevails in behavioural and evolutionary research (but see [75]). By choosing many different males for mating, *A. femoralis* females may hedge their bets against poor tadpole deposition choices of single males (cf. [57]) or against inferior genetic contribution [76]. Comparative research in species with different parental systems will help us better understand the role male parenting has in the link between polyandry and female reproductive success.

In females, we found a link between personality and the number of mating partners: females with either lower exploration levels, or higher exploration and boldness levels mated with more males. Our path analysis shows that these findings were not affected by a potential link between personality and female fecundity since there was no influence of any personality trait on the number of clutches in females. We assume that bolder and more explorative females rather mated with males further away, while less explorative females mainly selected males in their immediate surroundings.

In males, aggression and exploration levels influenced the number of mating partners, and indirectly influenced the number of clutches they sired. Males who obtained high numbers of mating partners were either non-aggressive and non-explorative or highly aggressive and explorative. On the one hand, less aggressive males, who might not be as competitive against intruding males could benefit from showing high presence in their own territory instead of exploring the area. On the other hand, more aggressive individuals who defend their territory fiercely might not be as good at distinguishing between potential mates and competitors and may thus even attack approaching females (M.R. and M.P., personal observations; see [77] for erroneous attacks on non-calling frogs). In turn, males with high exploration levels might increase their mating success by increasing their chances of settling in locations where there are more females. In a previous study on the same population, indeed exploration- and boldness-related behaviours in males were positively linked to the number of females in the vicinity of their territories [37]. A similar link between exploration levels and reproductive success has recently been found in red junglefowl (*Gallus gallus*), where in female-biased groups males with highest exploration levels also had increased mating success [78]. All these findings suggest that being bold and explorative can provide access to more mating partners for both the advertising as well as the choosing sex, depending on the reproductive strategies and movement patterns of the species.

In males, the effect of aggressiveness and boldness on the number of adult offspring depended on the male's exploration level. More aggressive and bolder males, or less aggressive and shyer males, had more adult offspring when their exploration level was high. Males likely benefit from high levels of exploration and boldness by finding more or better water bodies for tadpole deposition [79]. Aggression and boldness levels did not impact the number of adult offspring in individuals with low exploration levels. These findings suggest correlational selection favouring particular combinations of behavioural traits [18], since being on the extremes of the aggressiveness and boldness axes only increased the number of adult offspring in males with high exploration levels. Previous studies in other taxa have already hinted towards the importance of correlational selection. For instance, in male stream water striders (*Aquarius remigis*) mating success is linked to levels of aggressiveness, activity, and social plasticity, but this effect was dependent on male morphology [17].

Offspring behaviour might also account for the effect of male personality on number of adult offspring. Variation in animal personality is at least partially determined by genetics [80,81] and in several species personality has a heritable component [82–84]. Realized heritability in great tits (*Parus major*) was reported to be 54% (±5%) for early exploratory behaviour [82], and boldness is highly heritable in burrowing owls [85]. If *A. femoralis* tadpoles inherit their personality traits from their parents, we would expect highly aggressive offspring to be better at accessing food as they develop or more likely to find a suitable territory once they become sexually mature. Very shy tadpoles and subadults might increase their survival by successfully hiding from predators during their development. Future studies should investigate if and to what degree personality is heritable in *A. femoralis*, and how this might affect offspring performance. Such information is particularly interesting in species with complex life cycles, where adult and larval individuals occupy diverse environments with potentially contrasting challenges.

Most studies to date have estimated personality effects on single processes. In our study, we show that behaviours that are positively linked to one component of reproductive success did not provide equal benefit to another component, and we show that these effects are different for males and females. Since our models were fitted with a Poisson or a negative binomial error distribution with a log link function, our estimates closely approximate selection gradients and indicate how phenotype affects relative reproductive success [63]. Consequently, we can infer that in males, their behavioural phenotype exerts more influence on the number of offspring surviving until adulthood than it does on the number of female mating partners obtained. In females, their behavioural phenotype did not affect as much the number of surviving adult offspring, but it affected the most the number of mating partners—and this effect was almost double that in males. Our models also allowed us to estimate the overall effect of each personality trait on the number of surviving adult offspring, which is the closest variable to the realized reproductive success in this season (electronic supplementary material, table S3). Given that all other traits are average, boldness had the strongest effect on the number of adult offspring in males, while it was exploration in females.

Sex-specific evolutionary responses to selection depend on the reproductive strategy of the species and the shared genetic architecture of the phenotypes [86,87]. In the presence of sex-specific selection patterns, the evolutionary dynamics of the behaviours will depend on the cross-sex genetic correlation. When selection favours opposite types of phenotypes for males and females and the cross-sex genetic correlations are strong and positive, sex-specific evolution of behaviour will be constrained. In contrast, negative cross-sex genetic correlations may accelerate the rate of evolutionary divergence in the presence of antagonistic selection [86]. The amount of sex-specific genetic variance and the strength of the genetic covariance between the sexes will thus influence how sex-specific behaviour can evolve [88]. Studying the cross-sex genetic correlations of the behaviours in this population will shed light on the evolutionary potential of sexual dimorphism in behavioural traits.

In conclusion, our results suggest a potential effect of correlational selection and support the hypothesis that individual behavioural consistency and consistent between-individual differences are maintained through life-history trade-offs. This link could further lead to the evolution of different reproductive strategies.

## Data Availability

The datasets and code generated during and/or analysed during the current study are available in the Open Science Framework repository [90]. Supplementary material is available online [91].
